# 

**Published:** 1917-10

**Authors:** 


### ANNOUNCEMENTS

### ORAL AND DENTAL SURGEONS FOR THE ARMY

1. It is the present intention of the surgeon general to place in certain base, evacuation, and recovery hospitals a section of head surgery which is to include a division of plastic and oral surgery, to have charge of injuries and diseases of the mouth and essentially related parts.

2. This division of plastic and oral surgery shall, unless otherwise specified in individual instances, have charge of injuries and surgical diseases of the mouth and its essential structure including the bony framework and soft tissues of the face, and also of the neck when the major part of the injury is situated above the clavicle, with the exception of those diseases and injuries that properly come under the specialties of ophthalmology, oto-laryngology, neurologic surgery and diseases of the thyroid gland.

3. It is the intention of the surgeon general to place in charge of these units oral surgeons who are licensed to practice medicine, surgery and dentistry, who are willing to serve, and who have been accustomed to doing general major surgery of the face and neck, as above outlined.

4. There are not sufficient of these latter to make up the estimated number of plastic and oral surgery units required.

5. To make up for this deficiency, each of the remainder of these units will be in charge of a general surgeon especially deft at plastic and bone surgery, with a dental oral surgeon as his associate.

6. There are a great many dental oral surgeons in this country who have not a medical degree but have studied oral pathology and understand the mechanics of fractures, etc., although they have never done major surgical operations of the type that this war calls for, where minor surgical cases are the exception rather than the rule. Putting a dental oral surgeon as an associate to a general surgeon will make a well-rounded, plastic and oral surgery unit and will give to the dental oral surgeon an opportunity to assist in the major operative surgery of the face and neck as well as to do the more properly oral work.

7. Will you kindly suggest the names of oral surgeons, and dental oral surgeons whose training, practice and skill fit them for taking up the work outlined in paragraphs "3" and "6," giving a terse opinion of the abilities of each?

8. An early, carefully considered reply is urgently requested.—J. P. BLAIR, Joint Committee on Head Surgery, Council of National Defense, Washington, D. C.

---

### CONSERVING OUR DRUGS

One of the purposes of the Pennsylvania Public Safety Committee is to cooperate with the various industries throughout the state and the nation in conserving our resources. The drug and chemical trades have been hard hit by the war and the prices of medicines have in some cases become prohibitive. It is therefore felt that immediate measures should be taken to eliminate waste in the handling of pharmaceutical and biological products. It is well known that pharmacists as a rule are careful in handling these products. Yet carelessness is apt to crop up here and there and bring about waste.

We wish to bring to your attention the enclosed abstract of a paper entitled, "Conserving Life by Eliminating Waste," by Dr. R. P. Fischelis, Secretary of the Pennsylvania Pharmaceutical Association.

It is time to sound a warning to pharmacists, hospital authorities, physicians, dentists, veterinarians and all others engaged in manufacturing, supplying, dispensing and using drugs and biological products, that unless efforts are made on the part of all to eliminate waste through carelessness, deterioration or misapplication, we may be confronted with a serious situation regarding supplies of many drugs, chemicals and biological products most necessary for the conservation of life.

The demand for certain drugs and biological products, particularly for the large armies which are being raised, is bound to be unprecedented. The civilian population will need the same medical attention as it is accustomed to in time of peace, and patriotism demands that our boys at the front shall not be inconvenienced for lack of medical supplies.

Unless waste is eliminated in the handling of drug products, and remedies are more judiciously employed, shortages are bound to occur. Foreign governments have commandeered drugs in their countries from time to time when acute situations arose. Our country will be forced to do the same thing unless the professions demonstrate that they can handle the problem adequately themselves. Surely we do not want a dictator in pharmacy nor do we want situations to arise which will compel the government to further regulate business.

Such steps are inevitable, however, unless concerted efforts to conserve supplies of pharmaceutical and biological products are put forth. There is an inexcusable waste of biological products each year, due mostly to careless ordering on the part of the retailer. Let us stop and consider for a moment that if every drug store in the United States were to return but one package of diphtheria antitoxin to the manufacturer because it had become outdated and therefore useless, approximately 50,000 packages of this valuable remedial agent would be wasted—and this while lives are being lost elsewhere for want of the product. This is but one example, and when we take into consideration that there are 150,000 physicians and 15,000 veterinarians in addition to the 50,000 druggists in the United States, who use hundreds of biological products, the wastage possible, because of careless ordering, at once assumes enormous and startling proportions. Yet such a waste would occur if a majority of the members of the professions did not stop to contemplate the results of such carelessness.

All of us must stop thinking merely as individuals and consider the significance of multiplication of individual wastefulness and carelessness. A single package of any article, subject to deterioration, which becomes useless due to overstocking seems trivial, but when multiplied by thousands this trivial waste soon assumes formidable proportions. It makes no difference whether the pharmacist bears the loss in permitting an article to deteriorate or whether the manufacturer makes an allowance, there is nevertheless always a loss. And added to the loss of the product itself there is the loss of accessories like rubber, metal, glass, wood, paper, dyes, other chemicals, time, labor and money used in putting it up, which in these days are very expensive and in some cases rare commodities.

Of course, it is impossible to foretell with absolute accuracy what the demand for a certain perishable product will be, but the careful pharmacist can gauge demands pretty accurately and waste can be reduced to a minimum by careful study of conditions. It is unnecessary, particularly at this time, to order more biological or pharmaceutical products than are needed for use in the immediate future, as supply stations of manufacturers are now so conveniently located to every section of the country that in the case of epidemics, supplies of biological products, etc., can be obtained anywhere within twenty-four hours at the very latest. It is better to take advantage of supply facilities than of the privilege of returning goods. The former is economy; the latter is waste.

Overstocking of supplies of all kinds in the drug store is exceedingly bad practice from a commercial point of view as well as from the standpoint of national necessity just now. The practice of hoarding supplies of products which are apt to become scarce is also a poor one, from the point of view of the shrewd business men, aside from any moral consideration, owing to the uncertainty of market conditions and the uncertainty regarding the length of the war. It not only has the effect of inflating prices, but it may also serve as a boomerang and leave high-priced stocks on the hands of the retailer when normal conditions are restored.

The purchase in bulk of pharmaceuticals subject to deterioration is a wasteful procedure unless there are immediate prospects of disposing of them. It should always be remembered that quick turnovers bring greater profits than "free goods" lying on the shelves for long periods.

We must not overlook the fact that every pint of fluid-extract and every package of bacterin or serum manufactured represents materials more and more difficult to procure, as well as time and labor, which, unless properly utilized, represent absolute waste.

"Doing your bit" means more than flying the American flag over your store. It means enlisting actively in the work of *Conserving Life by Eliminating Waste.*

You are well aware that such products as the antitoxins and serums, including diphtheria antitoxin, tetanus antitoxin, antipneumococcic serum, antimeningitis serum, antistreptococcic serum; the serobacterins and bacterins, including those for acne, cholera, influenza, gonorrhea, pertussis, pyorrhea and typhoid fever; as well as smallpox vaccine, hay fever pollen extracts, tuberculins, Bulgarian bacillus, etc., are subject to deterioration, and when they are once outdated are of no use whatever. It is, therefore, essential that these products be not stocked in excess of reasonable requirements.

You also know that many galenical preparations are subject to deterioration and that this is particularly true of fluid-extracts and other preparations of ergot, digitalis and strophanthus. These preparations should therefore be ordered in sufficient quantities to last for not longer than six to twelve months. Unless they will be used in large amounts, they should not be ordered in bulk, and they should be kept in tightly stoppered containers. The same care in ordering and preventing waste should be exercised with all chemical and drug products.

May we not caution you to heed these warnings regarding the matter of overstocking? Everything you do as an individual ultimately affects the community at large, and the more you do to eliminate waste, the greater will be the result from our combined efforts in conserving life.—GEO. E. NITZSCHE, Executive Secretary, Department Sanitation, Medicine and Red Cross.

---

NATIONAL DENTAL ASSOCIATION.—Transportation arrangements to Twenty-First Annual Session, New York, N. Y., October 22-26, 1917. Those who are going to New York for the National Dental Association meeting October 22 to 26, are urged to consult the local ticket agent at their home town for complete information regarding fares, time limits and stop-over privileges in connection with the trip. There is on file in every ticket office the tariff schedule which officially informs local agents of the rates from that point.

You should not leave the determination of the route desired until just before you are ready to start on the trip. Local ticket agents may have to obtain special tickets for this occasion from their general offices, and you must therefore notify them in time so that the tickets will be available when you are ready to commence your journey.

From various points in the United States, passenger associations have announced special rates as follows: Chicago, Ill., $36.30; Cincinnati, Ohio, $30.10; Cleveland,

Ohio, $22.90; Columbus, Ohio, $25.30; Dayton, Ohio, $28.10; Detroit, Mich., $26.50; Indianapolis, Ind., $32.50; St. Louis, Mo., $42.20; Toledo, Ohio, $26.50.

This will be one of the greatest dental meetings ever held. Every one who can, should attend.

---

THE JOURNAL OF MILITARY DENTAL SURGEONS.—This is a new and surprisingly good and useful addition to our current literature. The September issue contains much very interesting and instructing information as to the relations of the dental surgeon to his comrades in arms, as well as a lot of information on the army dentist and what he is accomplishing among the soldiers, for humanity. The Journal is edited by Captain R. E. Ingalls, and is published quarterly by the Association of Military Dental Surgeons of the United States, at 323 Geary Street, San Francisco, California. It is very good reading and we wish it all success.

